# Ethical Implications of Alzheimer’s Disease Prediction in Asymptomatic Individuals through Artificial Intelligence

**DOI:** 10.3390/diagnostics11030440

**Published:** 2021-03-04

**Authors:** Frank Ursin, Cristian Timmermann, Florian Steger

**Affiliations:** Institute of the History, Philosophy and Ethics of Medicine, Ulm University, Parkstrasse 11, D-89073 Ulm, Germany; cristian.timmermann@uni-ulm.de (C.T.); florian.steger@uni-ulm.de (F.S.)

**Keywords:** autonomy, justice, population screening, preclinical, pre-symptomatic, machine learning, explicability, informed consent

## Abstract

Biomarker-based predictive tests for subjectively asymptomatic Alzheimer’s disease (AD) are utilized in research today. Novel applications of artificial intelligence (AI) promise to predict the onset of AD several years in advance without determining biomarker thresholds. Until now, little attention has been paid to the new ethical challenges that AI brings to the early diagnosis in asymptomatic individuals, beyond contributing to research purposes, when we still lack adequate treatment. The aim of this paper is to explore the ethical arguments put forward for AI aided AD prediction in subjectively asymptomatic individuals and their ethical implications. The ethical assessment is based on a systematic literature search. Thematic analysis was conducted inductively of 18 included publications. The ethical framework includes the principles of autonomy, beneficence, non-maleficence, and justice. Reasons for offering predictive tests to asymptomatic individuals are the right to know, a positive balance of the risk-benefit assessment, and the opportunity for future planning. Reasons against are the lack of disease modifying treatment, the accuracy and explicability of AI aided prediction, the right not to know, and threats to social rights. We conclude that there are serious ethical concerns in offering early diagnosis to asymptomatic individuals and the issues raised by the application of AI add to the already known issues. Nevertheless, pre-symptomatic testing should only be offered on request to avoid inflicted harm. We recommend developing training for physicians in communicating AI aided prediction.

## 1. Introduction

The World Health Organization estimates that in 2050 three times more people will be affected by Alzheimer’s disease (AD) than today and demands the development of early diagnosis [1,2]. Screenings are considered suitable for this purpose. To cope with the enormous financial and time-consuming effort of population-wide screenings, a two-step strategy is currently being tested. Both steps apply machine learning (ML), a subset of artificial intelligence (AI): first, identification of high-risk individuals on the basis of socio-demographic or prescription data [3,4]; second, individual biomarker assessment in high-risk individuals to identify eligible subjects for clinical trials with the future prospect of delaying or alleviating the progress of AD in affected persons [5,6].

The technology of AI aided AD prediction is already at an advanced stage. The first clinical AI decision support tool for predicting the progression from early-stage dementia to AD was recently tested in a multicenter study [7]. The trend leads to train ML systems on multi-feature datasets instead of assessing only one biomarker [3]. In addition to genetic testing and detection of biomarker sets [8], neuroimaging is used most often in conjunction with AI [9]. A recent model predicts AD 75.8 months prior to final diagnosis by using neuroimaging and performs better than radiologic readers [10]. Accuracy, sensitivity, and specificity when classifying patients with AD from healthy controls achieves almost the maximum [11].

Predicting the future onset of AD is accompanied by ethical challenges since there is no disease modifying treatment available yet [12,13,14]. Nevertheless, early diagnosis of prodromal AD is considered crucial for the well-being of individuals and society. Therefore, an international expert group demanded guidelines for risk assessment in asymptomatic subjects that can guide decisions on the application of screenings [15]. Although this topic is relevant for all countries world-wide, little has been done in this regard. We will give an example from Germany, because it is a result of major stakeholder consultations involving a wide range of medical specialties, we are most familiar with the local situation, and want to make it accessible for a non-German readership. In 2018, the German Medical Association (GMA) issued a statement on the use of predictive tests for the risk assessment of AD [16]. The GMA does not recommend offering predictive tests to asymptomatic individuals that have no familial AD background. Unfortunately, there is no justification for this conclusion except: (a) lack of evidence on the validity of predictive tests and (b) the challenges of informed consent. With the advent of AI aided AD prediction in asymptomatic individuals, the matter becomes even more urgent, as Eric J. Topol warns, otherwise a prominent supporter of medical AI [17]. Another multi-stakeholder project just underway is exploring the ethical challenges of dementia prediction in Germany, but does not address AI aided prediction [18]. This invites us to explore the ethical arguments related to AD prediction through AI and highlight their implications.

Although there is a large intersection with traditional biomarker and genetic assessment, the application of AI raises new ethical questions [19]. “Brain checks” for asymptomatic individuals are especially ethically challenging due to the lack of a medical indication [18]. A survey of the practice of AD prediction in 108 German hospitals shows that biomarker-based risk assessment is already used in clinical practice [20]. Before AI aided AD prediction is used for population-wide screenings as commentators demand [4], it must be considered under which circumstances it should be offered to asymptomatic individuals given the chance that there are unintended negative consequences, which possibly exceed the potential benefits for individuals and society. Similar ethical issues emerged in the discussion about incidental findings in the case of genetic risk assessment [21,22,23]. This debate can inform AI aided AD risk assessment. Incidental findings cannot be considered as unexpected [24], as diagnosis is by nature explorative and arrangements need to be made to provide adequate patient communication according to bioethical principles. Thus, considering informed consent as meeting the demands for transparency and minimizing foreseeable burdens are also crucial for the ethical assessment of AI aided AD prediction [25]. Another debate from which our discussion can benefit are the ethical guidelines for pre-symptomatic testing for Huntington’s disease, which have been used as an ethical model for genetic testing for early-onset AD [12,26,27,28].

Pending questions can be discussed within the ethical framework of the four bioethical principles: respect for autonomy, beneficence, non-maleficence, and justice [29]. We show that this framework adds value to the discussion of the clinical application of AI aided prediction. However, there is the new challenge of explicability that is specific to AI/ML algorithms: due to technological peculiarities, physicians cannot explain to the patient why the black-box algorithm predicts a severe illness, because of the algorithm’s opacity. This has a significant impact on obtaining informed consent. Although explicability is a major issue, recent reviews on the ethical and social implications of using predictive modeling for AD found that it is not covered in the literature [19,30,31]. Our approach differs from previous research on ethical implications of predictive modeling for AD as we systematically discuss the demands and challenges of explicability in medical AI in relation to the four established principles of biomedical ethics.

The new situation with the advent of medical AI does not obviate the already known challenges for biomedical ethics; we identified a large intersection between previously known ethical issues and those brought by the introduction of medical AI. In this paper, we will focus on the interaction between asymptomatic individuals and physicians. This interaction is the subject of our ethical analysis that aims to explore the arguments for and against offering AI aided AD prediction to asymptomatic individuals and their ethical implications. With this, we contribute to the ongoing discussion on AD prediction by offering an extensive assessment of the ethical landscape and highlighting the need for future research on ethics and policy-making.

## 2. Materials and Methods

**Figure 1:**
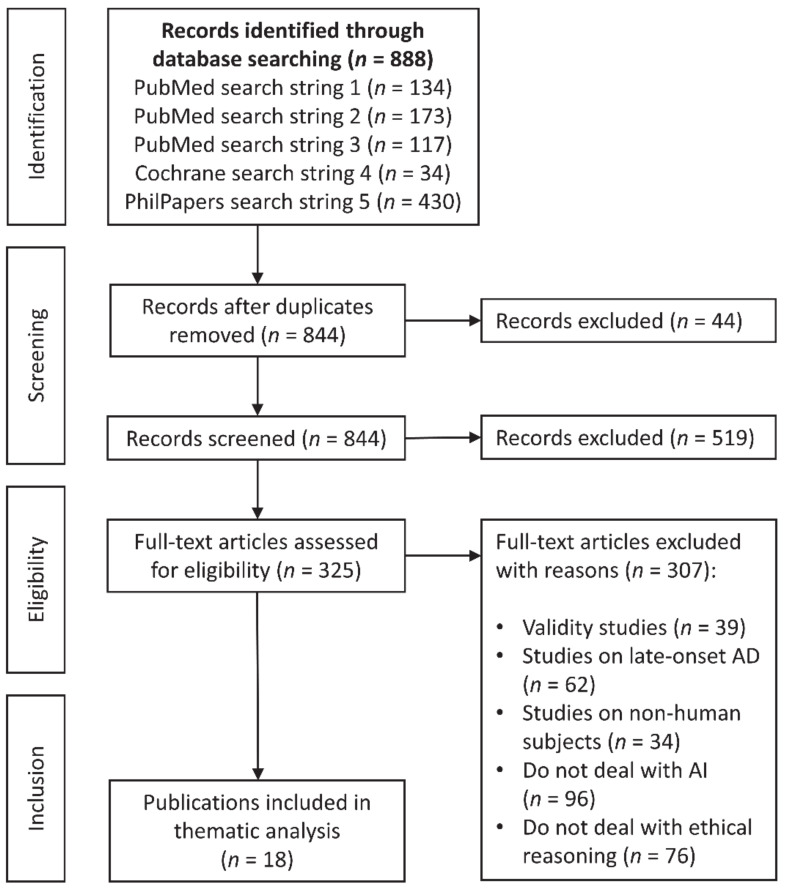
Flowchart of bibliographic database searching resembling the PRISMA (2009) statement.

The assessment of the ethical arguments for and against AD prediction through AI is based on a systematic literature search. The systematic search was conducted according to the PRISMA statement as indicated in Figure 1 [32]. The included publications were examined by a thematic analysis resembling the method of “systematic review of reasons” [33]. This method consists of four steps: (1) Formulation of the review question and eligibility criteria; (2) Identification of literature that meets the eligibility criteria; (3) Extraction and synthesis of the data; (4) Deriving and presenting results to answer the review question. We have slightly adapted this method to fit our research question: Which ethical arguments are used in favor of or against offering AI aided AD prediction in subjectively asymptomatic individuals?

An initial search in PubMed with the search string ((“artificial intelligence”) OR (AI)) AND (alzheimer) AND (ethic*) yielded no significant publications (*n* = 76). Therefore, we searched systematically in three bibliographic databases with five search strings to obtain publications that elaborate, review, discuss or evaluate ethical arguments. The mix of the databases PubMed, Cochrane Library, and PhilPapers was chosen on the grounds of obtaining both clinically and ethically relevant arguments. The last search was conducted on 19 November 2020. The eligibility criteria for including publications are: conceptual studies, opinion pieces, qualitative research, original articles, and reviews in peer reviewed journals. Publications in English, French and German language were included without restrictions on publication date.

First, we gathered publications by a search in PubMed with search string 1 (“Alzheimer Disease/diagnosis” [MeSH] AND “Ethics” [MeSH]) (*n* = 134). To complement this search with less restrictive keywords, we secondly searched PubMed with the search string 2 (alzheimer*) AND (early diagnosis) AND (ethic*) (*n* = 173). Third, to provide an empirically grounded ethical analysis the evidence of the present diagnostic power of AI aided AD prediction was collected through a search in PubMed with the search string 3 ((artificial intelligence) OR (deep learning) OR (machine learning)) AND ((Alzheimer’s disease)) AND ((prediction) OR (diagnosis)) AND (review) (*n* = 117). Fourth, Cochrane Library has been searched with search string 4 “Alzheimer” AND “diagnosis” within titles, abstracts, and keywords (*n* = 34), but there was no systematic review yet on early diagnosis or prediction through AI. Fifth, to gain more ethical and philosophical publications, we searched the bibliographic database PhilPapers with the search string 5 “Alzheimer” (*n* = 430).

The searches yielded *n* = 888 records. A total of *n* = 844 were screened for titles and abstracts after duplicates were removed. Exclusion criteria for eligibility were as follows: validity studies on specific measures or tests (*n* = 39), studies on late-onset AD (*n* = 62), studies on non-human subjects (*n* = 34), studies that do not deal with AI (*n* = 96), and studies that do not deal with ethical arguments (*n* = 76). A total number of *n* = 18 eligible articles were included in a thematic analysis that allowed to answer the research question. Thematic analysis was conducted inductively by one reviewer to identify, analyze, and report recurrent patterns of arguments in included publications [34,35]. The initial coding was conducted by F.U. and reviewed by C.T. and F.St. The pooling of the data was carried out in two steps: first, identified arguments have been assigned to overarching themes until the level of abstraction was sufficient; second, reasons have been binary assigned to pro and contra arguments. This process was conducted by F.U. and reviewed by C.T. and F.St. until consensus was reached. The authors of the article have a background in medicine, ethics, philosophy, and history of medicine. Because this study was interested in the relation between asymptomatic individuals and physicians, the pooling of the qualitative data was conducted to address ethical implications both from an individual and a societal perspective.

## 3. Results

Examined publications include original contributions, questionnaire surveys, focus group studies, ethical evaluations, a strategic road map for research, and reviews (Table 1.). To some extent the ethical evaluation depends on use case, because rights and duties may vary depending on the use context for the involved moral agents (Table 2.): e.g., researchers towards participants (research ethics); physicians towards patients in clinical treatment (clinical ethics); governments towards citizens in society (public health ethics). The use cases have been built by pooling additively from different publications, because not all use cases were mentioned in one publication. We present an extended list of ethical arguments for and against offering AD prediction to asymptomatic individuals in Table 3 and Table 4. In what follows, we clustered the most common ethical issues into three groups to facilitate a better overview: (a) individual benefits and disadvantages, (b) social benefits and disadvantages, and (c) right to know vs. right not to know.

### 3.1. Individual Benefits and Disadvantages

Benefits of AD risk disclosure can derive from resolving the uncertainty of one’s own AD risk if it brings clinically meaningful information and the subject is willing to know the risk [19,31,37]. Benefits can also derive from future planning in regard to potential caregivers, housing situation, family life, adjustment of insurance, financial planning, and end-of-life care [19,20,31,37,38,40]. Promotion of research and improved control of the disease’s progression are seen as benefits [19,20,38]. There is the opportunity to change unhealthy lifestyles [37,41]. In case of symptomatic patients actively seeking support in a research setting, the benefits of early detection outweigh potential adverse effects [37].

A reason against offering predictive testing is the lack of a disease modifying treatment. Knowing one’s risk does not alter the disease [19,30,31]. A general screening (second use case in Table 2) is only considered individually useful if effective treatment options are available [19,20]. Predictive testing is held to be ethically acceptable only in research (first use case in Table 2) because the predictive value is unclear and preventive measures are not available [19,38].

Without disease modifying treatment the risks of a disclosure are potential psychosocial harms, distress, anxiety, remaining post-testing uncertainty, possible false-positive or false-negative diagnoses, stigmatization (public stigma, self-stigma, spillover stigma), and discrimination in health insurance and at work [19,20,30,31,38,40,41,45,46]. Since a disclosure also affects the family and potential caregivers, unanswered questions remain regarding social burdens like isolation, discrimination, and social rejection [40,41,45]. Patients may consider a “rational suicide” based on financial reasons and to reduce family burden [30]. It is unknown how much support an individual needs after learning about a diagnosis of preclinical AD [45]. In case of asymptomatic volunteers who are tested in a research setting, the potential adverse effects overweigh the benefits and therefore a restrictive disclosure policy should be applied according to the “principle of caution” [37]. It is considered unethical to disclose biomarker results to asymptomatic patients attending a memory clinic [39].

### 3.2. Social Benefits and Disadvantages

The cost-effectiveness of both predictive tests and future hypothetical preventive treatment is considered a requirement for offering predictive tests, but opinions are divided regarding whether tests would save public health resources or increase costs [19,20,31]. Tests outside of a research setting that are used for profit in individuals for whom a risk assessment is not indicated should be limited [15]. Policy-makers may, however, have an interest in performing random tests or general screenings to gather information about changing trends in order to facilitate long-term planning. Here the interests of individuals and policy-makers may conflict [4].

In terms of social rights, there are no international agreements on the protection of subjects that underwent a biomarker test as in the case of genetic privacy [36,45]. Regulations to protect social rights and privacy are demanded, because worries exist that health insurances deny coverage or charge higher premiums if a diagnosis for AD is in the medical record [19,20,30,36,45]. Further concerns regard the protection against discrimination in employment, exclusion from medical decision making, or the withdrawal of a driving license without an adequate alternative to guarantee mobility [3,20,30,45,46].

### 3.3. Right to Know vs. Right Not to Know

There are significant differences in personal preferences in undergoing predictive testing [42]. People have the right to know the risks, but respect for autonomy requires a physician to determine whether or not an individual wishes to receive disclosure [19,30,38,41]. Offering tests and disclosing test results is in most cases considered to be in accordance with the respect for the autonomy of a person and a way of empowerment [37,41]. Reasons for wanting to know one’s biomarker status are the need for clarity, informing family members, and planning for the future [40,43].

The right to know stumbles against two major obstacles: the accuracy of diagnosis and the (in)explicability of results. Although the accuracy of predictive AI systems has significantly increased in recent years [3,9,44], there is no consensus about which predictive power is sufficient [31]. A poor sensitivity (false-negative diagnoses) might lead to false reassurance and exclusion from treatment or clinical trials; a poor specificity (false-positive diagnoses) might lead to over-diagnosis, over-treatment, inappropriate inclusion in clinical trials, and invasive biomarker testing that can be harmful [12,15,31,37]. Overall, the amyloid cascade hypothesis, on which biomarker assessment rests, is contested in research [38].

Persons undergoing testing may not understand its predictive value and are subject to therapeutic misconception [30,40]. Communicating an AD diagnosis or risk assessment is considered challenging due to different degrees of understanding of the disease and the uncertainty of preclinical risk assessment [12,30,31,40]. Interview studies show that persons who have learned their elevated biomarker level also understand that their risk of developing AD is increased but uncertain, however, they wished to know how a dimensional biomarker was turned into the categories “elevated” or “not elevated” [45]. This wish cannot be met with AI, because these black box systems only distinguish between “has that condition” or “does not have that condition” [3]. Therefore, an explainable AI is demanded that is transparent in its “decisions” [3]. Further challenges are accountability, algorithm bias due to insufficient training data, application of passive surveillance tools, and regulatory approval [3]. Challenges are seen in the paradigm shift to so-called “desktop medicine”, where individuals learn about their health from test results and not based on symptoms [31].

There are also strong arguments for supporting a right not to know. Reasons for not wanting to know one’s biomarker status are anxiety about AD and that disease modifying treatments are not yet available [19,30,40,41]. According to a survey in German hospitals, 81% of physicians acknowledge the patient’s right not to know of the individual biomarker status, but disclosure practice shows that 75% of physicians do always communicate results of predictive testing [20]. Furthermore, 10% communicate results only on request and 20% communicate results only in combination with psychological consultation [20]. On the other side, a culture of withholding dementia diagnosis is described which argues in favor of non-maleficence [30]. This may have adverse effects in terms of being paternalistic, although a physician may have good intentions by withholding information [37]. Study participants have the ethical right to a full disclosure upon request based on the principle of autonomy [19,37].

## 4. Discussion

We discuss the ethical implications of AD diagnosis in relation to the four bioethical principles. The discussion of the respective arguments in Table 3 and Table 4 is assigned to one of the following four sections, each addressing a bioethical principle. In the cases where an argument can be discussed under more than one principle, we opted for the principle that facilitates the most accessible description of the ethical dilemmas. Under the perspective of autonomy, we discuss arguments regarding the right to know vs. the right not to know, elements of informed consent, adequate counseling when AI is involved, and the need for explainable AI. Under the perspective of beneficence, we discuss arguments of improved precision and reduced human errors, but also the practical hindrances of implementing AI in the diagnostic process. Under the perspective of non-maleficence, we discuss the harms that can be caused by AD prediction, threats of automation bias and algorithmic bias, and the question of liability of black box systems. Under the perspective of justice, we discuss arguments concerning access to healthcare, distributive justice, arguments concerning cost-effectiveness, and the tensions between individual and societal needs.

To complement the discussion of our results, we deem it helpful to consider the already developed ethical guidelines for pre-symptomatic testing for Huntington’s disease. These can be used as an ethical model for genetic testing for early-onset AD [12,26,27,28] and therefore can serve as discussion basis for AI aided AD prediction. These guidelines foresee that [47]:

1. The test should be voluntary and based on informed consent.
2. The test should be offered with proper counseling and professional support.
3. The test should only be available to mature adults.
4. The test results should not cause discrimination.
5. Testing should be delayed if there is evidence that the results will lead to psychosocial harm.
6. The test results are confidential and the property of the individual.

Further insights are gained from the literature on explicability.

### 4.1. Autonomy

We found that there is a discussion about whether offering predictive testing to preclinical individuals may violate their autonomy [30,46]. The literature distinguishes between two scenarios: a patient who autonomously requests a test, and one who is offered a test although he does not request it or show any desire to acquire such information [48]. Patient’s autonomy may be violated, if he is involuntarily tested or informed about the test results [49]. Respect for the patient’s autonomy includes the negative duty not to impose unwanted information and the positive duty to disclose information and actions that foster decision making [50]. Patient’s autonomy can be respected by informed consent and can be violated by withholding pertinent information or ignoring a refusal of medical intervention or diagnosis.

We found that the right to know is a central argument in favor of predictive testing [19]. The right to know is a prerequisite to allow individual choice for patients, to respect individuals as autonomous subjects, and to facilitate future planning. Conversely, the right not to know has originally been justified to defend a person’s interest in not spoiling a certain element of surprise, for example concerning the gender of one’s offspring, or more recently to allow the reasonable enjoyment of certain foodstuffs without the imposition of details of its production or content [51]. In our case, the right not to know is a response to the predictive power of new diagnostic methods, which predict a risk of serious disease in a context where no effective treatment is available [19,30]. It is an exception to the physician’s duty to inform [52]. Obtaining assent to predictive testing and disclosing of the results honors the autonomy of patients and involves them in the decision process [53]. For various reasons, a patient may not want to know the result of a test even if he initially asked for it, because, e.g., family members or potential caregivers are often present when disclosing a diagnosis [54]. A patient’s values and wishes should be the primary determinant for disclosure decisions, whether they coincide with their caregiver’s wishes or not [53].

Empirical data show that most people want to know whether or not they will develop AD [55]: more than half (56.1%) want brief information and immediate referral to a family practitioner or a specialist. Only 9.8% want no information because they claim to have a right not to know. These results concur with earlier evidence according to which most patients want to know the truth about their dementia diagnosis, although they feel upset after disclosure [56]. At the same time, these results clearly indicate that physicians should not assume that all patients want to receive such information. Two provisions apply: first, consistent with the principle of beneficence, the physician may educate the patient about the benefits of advanced planning for autonomy in the event of becoming incapacitated [29]. Second, the right not to know should not be used to shield patients from knowledge about the existence of such diagnosis possibilities [57]. Information generally supports autonomy. A failure to inform about such tests may jeopardize future planning capacities to protect an interest that can be considered as of second order.

Another determinant for disclosure decisions is the psychological state of the patient. It has been argued that postponing diagnosis disclosure is acceptable if the patient is in a bad psychological state [58]. In accordance with the principle of non-maleficence, the physician has to consider the patient’s cognitive and social resources as well as psychological coping skills [53]. We argue that postponing diagnosis disclosure is ethically appropriate if the principle of non-maleficence weighs heavier than the respect for temporary autonomy at the physician’s reasonable discretion. A test may not be in the patient’s best interest in times of strong distress since the results would not change treatment or outcome [28]. A physician’s duty to immediately warn a patient in order to prevent harm may not apply, because the disorder is inevitable and no disease-modifying treatment is available.

AI assisted diagnosis suggests an enormous challenge regarding the informing of patients about results and future action. The limitations of the test results must be interpreted and communicated to the patient, especially regarding the accuracy, sensitivity and specificity of the given AI aided diagnostic tool [30]. An emerging aspect when applying AI aided diagnosis for preclinical patients is a new type of counseling, which is comparable to traditional genetic counseling [23]. Patients should be informed via “digital counseling” about the AI system that was involved in the process of prediction, risk assessment or diagnosis. There are promising attempts to overcome such communication challenges by assisting the information process with graphics. For instance, explainable convolutional neural networks as diagnostic tools for early detection of AD are being developed by visualizing certain patterns [3,10,59]. Activation maps point to the relevant areas in neuroimaging data and thereby can provide intuitive understanding of model output for clinical users. A simplified and visually enhanced version of these maps can be shown to patients to facilitate the information process.

Protocols to safeguard patient autonomy and privacy may slow down technology developments. The challenge is that machine learning algorithms can learn continuously when new data is included. Although learning systems are desired because they increase diagnostic accuracy, this may conflict with existing regulations for “Software as a Medical Device” that aim to restrict the transfer of sensible data [60]. Once the data is shared by both the training and testing sets, the new data influences future classification decisions [61]. Thus, a systematic and controlled process of data curation is needed for “Software as a Medical Device”. Technology developers and collaborating physicians may have a conflict of interests when it comes to technology optimization that needs to be communicated to patients to find out whether they still give informed consent to participate in this experimental optimization process.

We conclude that it is ethically appropriate to offer predictive testing to persons in case they ask for it, because this is not imposed information, as long as they are in a condition to assess the communicated information. Informed consent remains the guiding principle for both performing predictive testing and for disclosing results. Because informed consent includes voluntariness, competence to decide, information disclosure, and understanding by those receiving the information, all these aspects have to be secured in case AI is involved. However, currently there is an ongoing debate as to whether or not patients have to be informed that AI is involved in the diagnosis or treatment [62]. This question is still not settled and must be addressed by future legal and ethical inquiries.

### 4.2. Beneficence

As a general ethical guideline, early diagnosis of AD must be beneficial to the patient, otherwise the test should be avoided. Beneficence requires the prevention of harm, providing benefits, and balancing benefits against risks and costs [29]. There are several areas in which early risk assessment of AD may be beneficial toward patients: exclusion of other causes for cognitive impairment and planning for the future [63]. An early diagnosis enables patients and their families to understand the disease, to decide on emerging financial burdens, and to arrange for the future needs and care of patients [8]. To this end, the World Health Organization, Alzheimer’s Disease International and several national public health authorities advocate early diagnosis in their dementia action plans [12]. Extensive counseling and appropriate education have shown positive outcomes following predictive testing [64].

AI aided diagnostics could help reduce human errors. It is unfortunate that there might be a reluctance among physicians to use AI aided diagnostic tools on several grounds [3,65,66,67]: first, black box systems are not transparent in their “decisions”; second, there might be technical and financial barriers to integrate these systems into clinical workflow; and third, there is the need to ensure information privacy with proper reliable data labeling, because risk assessment produces sensible data. The principle of beneficence invites at least an exploration of AI aided diagnosis as a method to improve precision and reduce errors.

### 4.3. Non-Maleficence

The overall goal of the principle of non-maleficence is not to cause harm to patients by a medical intervention or diagnosis [29,50]. We found that predictive AD testing has been considered unethical because no effective prevention and disease-modifying treatment is available [19,68]. This holds true in the present research setting in which volunteers obtain disclosure of their risk assessment [37,39]. Disclosing a diagnosis for AD can cause harm to patients [19]: there is an increased risk of depression, psychiatric hospitalization, stigmatization [69], and suicide as in other neurological diseases with a not favorable prognosis [26,70], e.g., Huntington’s disease [71,72]. Additionally, potential consequences of learning information about AD risk on life and health insurance coverage, employment, driving license and implications for an individual’s social position and identity have to be considered [30,46]. Moreover, although there are lower suicide rates in the long run of diagnosed dementia patients compared to control groups with no neurological disorder, the death by suicide ratio is three times higher than in controls during the first month after diagnosis [70]. We conclude that it depends on the use case whether possible adverse effects of disclosing results of predictive testing overweigh the benefits of knowing one’s risk. Adequate counselling may mitigate negative effects and therefore needs to be part of the diagnosis procedure.

A physician who provides AD prediction through AI must be able to explain on which grounds the algorithm came to its decision. Since physicians are always responsible for a medical procedure [73], intelligibility is crucial, because diagnostic systems have to be explainable, interpretable, understandable, and transparent in terms of their decision-making properties [74]. In terms of accountability, liability is an unsolved challenge. In a joint European and North American multi-society statement on the ethics of AI in radiology, automation bias and liability of black box systems have been addressed as the most important ethical challenges [73]. Automation bias leads humans to prefer machine-generated decisions over those of humans. The question is to what degree physicians can delegate the task of diagnosing to autonomous systems without exposing themselves to increased liability for malpractice in case the system makes an error [75]. Thus, there is a need for different liability models for different use cases and for a risk liability system. The medical societies state that physicians will remain ultimately responsible for patient care [73]. The question arises whether AI aided diagnosis is a mere second opinion [76], and if it is, why it should be used given all its disadvantages such as costs of development and implementation, limited explicability and liability questions [65]. Advantages like cost-effectiveness once installed, time-saving, high accuracy, and the fact that they outperform physicians in diagnosing may lead to their implementation [77,78]. Commentators outline future prospects in which delegating time-consuming activities to autonomous systems enables physicians to spend more time on their core tasks [79]. To meet the principle of non-maleficence, the advantages of AI need to benefit the individual patients and overall lead to less hazards. An overemphasis on cost saving may leave responsibility issues unaddressed and undermine obligations to adequately inform patients.

### 4.4. Justice

Justice in our case refers to three distinct principles: firstly, to the principle that like cases should be treated alike, secondly, to distributive justice demanding a fair, equitable and appropriate distribution of health care in society [29,50], and thirdly, to assess whether our innovation system contributes to structural injustices by continuing to disadvantage historically marginalized groups [80].

Access to dementia diagnostics is limited by two types of resources. First, the number of physicians in the specialties of geriatrics, neurology, and geronto-psychiatry will be insufficient, because it is expected that the number of AD cases will increase [8]. Second, cost-effective and accurate diagnostic tools are needed. Since neuroimaging is expensive, cerebrospinal fluid analysis is invasive, and neuropsychological assessment is time-consuming, the cost-effectiveness of neuroimaging could be improved by AI [3]. However, there are worries about the disparities in access to AI aided dementia diagnostics since smaller hospitals and academic departments may lack the technology, skills, and resources to manage complex AI systems, or these systems are proprietary as they are developed by large academic or private health care entities [73].When the accuracy of prediction by AI aided neuroimaging outperforms cheaper diagnostic tools like assessment of speech, gait, blood, and electroencephalography, then its application should be considered. Efforts to expand access to AD diagnosis still need to keep in mind that providing adequate information and counselling is part of the diagnosis and should not be sacrificed under the excuse of making diagnosis more widely available.

There are ethical tensions between individual and societal needs when it comes to AD diagnosis. The limitation of health care resources does not permit AD diagnosis to satisfy mere individual curiosity. Conversely, while an individual may insist on the right not to know by referring to autonomy, from a societal perspective there are several reasons to know who may develop AD, some backed by the principle of justice. Insurance companies, lending banks, employers, participants in road traffic, as well as family and marriage partners may need to adapt their future behavior and policies [30,46]. Governments may need to carry out population wide studies to assess future health needs and thereby secure sufficient funding and health professionals for future needs. Massive refusal to test will, however, not allow governments to make the necessary arrangements for an eventual sharp increase in care cases which, particularly in welfare states, is their obligation. As long as citizens agree on guaranteeing the common good of universal healthcare coverage protection, they need to do their share in establishing such a good. This may include consenting to randomized governmental testing. When such tests are done, it is unclear whether or not identified at-risk individuals may be contacted: on the one side, there is an ethical conflict between respecting the right not to know and the potential harm of disclosure of being an at-risk subject. As these tests may disclose information that can be important to individuals, a minimum level of diagnosis disclosure is unavoidable to facilitate future planning. On the other side, there is the societal need for participants in clinical trials and drug development studies. We conclude that the respect for autonomy, the right not to know, and the principle of non-maleficence must be weighed with societal needs for knowing who will develop AD in the future.

## 5. Conclusions

From an ethical point of view, it is crucial that predictive testing is safeguarded by ethical principles which serve the needs both of individuals and society. In a given clinical case, the principles and respective arguments can guide decision making by weighing the principles against each other. At the individual level, the most important principle is the respect for autonomy, which can be secured by determining whether a subject has the wish to undergo predictive testing or not. As a general guideline and given the present technological, regulatory, and ethical circumstances, predictive testing should only be offered to asymptomatic individuals upon request in a research use case, which is protected and regulated by research ethics. The risk of causing psychosocial harm by disclosing test results must be as small as possible and the limits of accurate prediction must be communicated to whoever requests a predictive test. Despite the individual risk, governments need to inquire about the level of disease prevalence to make the necessary arrangements for future social welfare. The ethical dilemma is that the test produces clinically and socially useful information, while there is no disease modifying treatment. Therefore, the ethical appropriateness of population-wide screenings that apply AI/ML algorithms to identify at-risk individuals is questionable at present.

The future prospect is to focus on preventive strategies and to improve ethical training to provide proper counselling. Because we still lack international standards and guidelines, the literature advocates the need for structured training in communicating diagnoses, and guidelines for counseling about the risks and benefits of testing, as well as for disclosure practice. Because we found analogies in the literature to other neurodegenerative diseases, helpful guidelines may be developed by translating, adapting, and modifying guidelines for these conditions which can be diagnosed in an asymptomatic state. Counseling is considered crucial for the process of informed consent, the patient’s right (not) to know, and avoiding harm. This addresses the competency of neurological, geriatric, geronto-psychiatric, and radiological associations to develop standards for counseling on AI aided diagnosis. We still need research in what this means specifically in the case of AD prediction in asymptomatic individuals through AI.

We found that patients undergoing biomarker assessments want to know what it means to have an “elevated” biomarker status. Here, we also need more research concerning two ethical dimensions. Firstly, as a matter of transparency it has to be disclosed that AI systems are deployed for prediction. Secondly, respect for autonomy demands explicability. It is ethically necessary to develop explainable AI systems for future clinical applications. These two dimensions are crucial to build trust within the interaction between patients and physicians. Only if patients trust both the oversight of physicians and the robustness of AI aided prediction, then offering it to asymptomatic individuals can succeed. For future clinical application, we recommend the adaptation of ethical training for physicians in communicating AI aided prediction by drawing on the experience in diagnosing other neurodegenerative diseases. The training should incorporate the challenges of transparency and explicability in securing adequate informed consent.

## Figures and Tables

**Table 1:** Included literature on the ethical arguments for and against offering AI aided AD prediction to asymptomatic individuals.

Authors	Title	Type of Study	Date
Smedinga, Tromp, Schermer, Richard	Ethical Arguments Concerning the Use of Alzheimer’s Disease Biomarkers in Individuals with No or Mild Cognitive Impairment: A Systematic Review and Framework for Discussion	Systematic Review	2018
Vanderschaeghe, Dierickx, Vandenberghe	Review of the Ethical Issues of a Biomarker-Based Diagnoses in the Early Stage of Alzheimer’s Disease	Systematic Review	2018
Milne, Karlawish	Expanding engagement with the ethical implications of changing definitions of Alzheimer’s disease	Correspondence (conceptual)	2017
Whitehouse	Ethical issues in early diagnosis and prevention of Alzheimer disease	Original Article (conceptual)	2019
Vanderschaeghe, Vandenberghe, Dierickx	Stakeholders’ Views on Early Diagnosis for Alzheimer’s Disease, Clinical Trial Participation and Amyloid PET Disclosure: A Focus Group Study	Focus Group (qualitative)	2019
Erdmann, Langanke	The Ambivalence of Early Diagnosis—Returning Results in Current Alzheimer Research	Risk-Benefit-Assessment (conceptual)	2018
Schermer, Richard	On the reconceptualization of Alzheimer’s disease	Original Work (conceptual)	2019
Hughes, Ingram, Jarvis, et al.	Consent for the diagnosis of preclinical dementia states: A review	Review	2017
Davis	Ethical issues in Alzheimer’s disease research involving human subjects	Original Work (conceptual)	2017
Schweda, Kögel, Bartels, Wiltfang, Schneider, Schicktanz	Prediction and Early Detection of Alzheimer’s Dementia: Professional Disclosure Practices and Ethical Attitudes	Survey (qualitative and quantitative)	2017
Stites, Milne, Karlawish	Advances in Alzheimer’s imaging are changing the experience of Alzheimer’s disease	Narrative Review (conceptual)	2018
Angehrn, Sostar, et al.	Ethical and Social Implications of Using Predictive Modeling for Alzheimer’s Disease Prevention: A Systematic Literature Review	Systematic Review	2020
Ivanoiu, Engelborghs, Hanseeuw	Early diagnosis of Alzheimer’s disease (with the announcement of the diagnosis)	Original Work (conceptual)	2020
Mattsson, Brax, Zetterberg	To Know or Not to Know—Ethical Issues Related to Early Diagnosis of Alzheimer’s Disease	Original Work (conceptual)	2010
Frisoni, Boccardi, Barkhof, et al.	Strategic roadmap for an early diagnosis of Alzheimer’s disease based on biomarkers	Whitepaper	2017
Ebrahimighahnavieh, Luo, Chiong	Deep learning to detect Alzheimer’s disease from neuroimaging: A systematic literature review	Systematic Review	2019
Graham, Lee, Jeste, et al.	Artificial intelligence approaches to predicting and detecting cognitive decline in older adults: A conceptual review	Conceptual Review	2020
Gautam, Sharma	Prevalence and Diagnosis of Neurological Disorders Using Different Deep Learning Techniques: A Meta-Analysis	Systematic Meta Review	2020

**Table 2:** Use cases for offering AD prediction to asymptomatic individuals.

Use Cases	Ethical Assessments	Source
(1) research	protected by good clinical practice research frameworks, ethics approvals by ethics committees, and informed consent	[15,19,36]
(1a) symptomatic patients actively seeking support	benefits of early detection outweigh potential adverse effects	[37]
(1b) asymptomatic volunteers which consent to predictive testing	potential adverse effects overweigh the benefits, therefore restrictive disclosure policy according to the “principle of caution”	[37]
(2) screening everyone	“turning everyone into patients” and “patients-in-waiting”	[31,38]
(3) screening those with known risk factors	partial exclusion of patients and “patients-in-waiting”	[31,38]
(4) psychological screening before screening	partial exclusion of potential patients	[31]
(5) voluntary access to screening for everyone	considered ethically justified to disclose biomarker results on request	[31,39]
(5a) symptomatic patients actively seeking support	benefits of early detection outweigh potential adverse effects	[37]
(5b) asymptomatic patients actively seeking support	considered ethically unjustified to disclose biomarker results	[39]

**Table 3:** Ethical arguments for offering artificial intelligence aided Alzheimer’s disease prediction to asymptomatic individuals.

Themes	Argument	Source
Benefits	resolving uncertainty of one’s risk is beneficial when it brings clinically meaningful information and the subject is willing to know his risk or diagnosis	[19,31,37]
	knowing one’s risk enables future planning	[19,20,31,37,38,40]
	knowing one’s risk enables promotion of research and control of the disease’s progression	[19,20,38]
	knowing one’s risk enables change of unhealthy lifestyle	[37,41]
Right to know	good communication requires a physician to determine whether an individual wishes disclosure based on personal preferences	[19,30,38,41,42]
	respect for the individual’s autonomy and empowerment	[37,41]
	clarity, informing family members, and planning for the future	[40,43]
Slippery-slope-argument	voluntary screening is justified since commercial genetic testing for AD is already available	[31,36]
Economy	cost-effectiveness is a requirement for predictive tests and future hypothetical preventive treatment, but opinions are divided whether tests would save money or increase costs	[19,20,31]
	restriction of tests beyond research use cases that are used for profit in individuals for whom a risk assessment is not indicated	[15]

**Table 4:** Ethical arguments against offering artificial intelligence aided Alzheimer’s disease prediction to asymptomatic individuals.

Themes	Argument	Source
Lack of disease modifying treatment	knowing one’s risk or diagnosis does not alter the disease	[19,30,31]
	general screening is only considered useful if effective treatment options are available	[19,20]
	predictive testing is only seen as ethically acceptable in research because predictive value is unclear and preventive measures are not available	[19,38]
Accuracy	although predictive AI systems have a high accuracy, there is no social consensus about which predictive power is sufficient	[3,9,31,44]
	no clinical use before regulatory approval because of the risk of false-negative and false-positive diagnoses, no consensus about sufficiency of predictive power, amyloid cascade hypothesis is contested in research, patients may not understand the predictive value and undergo therapeutic misconception	[12,30,31,38,40]
	false-negative diagnoses may lead to false reassurance and exclusion from treatment or clinical trials	[15,31,37]
	false-positive diagnoses may lead to over-diagnosis, over-treatment, inappropriate inclusion in clinical trials, invasive biomarker testing can be harmful	[15,31,37]
Risks	avoidance of psychosocial harm because of distress, anxiety, remaining post-testing uncertainty, possible false-positive or false-negative diagnoses, stigmatization (public stigma, self-stigma, spillover stigma), discrimination in health insurance and at work	[19,20,30,31,38,40,41,45,46]
	avoidance of harm to third parties because of family burdens, social burdens (isolation, discrimination, and social rejection)	[40,41,45]
	avoidance of harm to subjects and third-parties because of “rational suicide” based on financial reasons and to reduce family burden	[30]
Right not to know	wish not to know because of anxiety and disease modifying treatments are not available	[19,30,40,41]
	avoidance of forced information because it violates respect for autonomy	[20]
Explicability	patient’s different degrees of understanding the disease and the uncertainty of preclinical risk assessment entail a challenging communication of diagnosis or risk assessment	[12,30,31,40,45]
	demand for the transparency of the “diagnostic decision” due to involvement of AI/ML (machine learning) systems and black box algorithms	[3]
	demand for governance models for patient’s data, data security, infrastructure for gathering and managing data, accountability, algorithm bias, passive surveillance tools, and regulatory approval	[3]
Threats for social rights	need for international agreements on the protection of subjects that underwent a biomarker test like in the case of genetic privacy	[36,45]
	worries that health insurances deny coverage or charge higher premiums	[19,20,30,36,45]
	worries about employment discrimination, exclusion from medical decision making, or the withdrawal of one’s driving license	[3,20,30,45,46]
Training	demand for structured training of physicians to counsel patients about AI/ML systems	[15,45]
Guidelines and standardization	demand for guidelines about information and disclosure practice	[15,20,36]
	demand for standardization of test methods, threshold values, data protection	[15,20]

## Data Availability

The data presented in this study are available within the article.
